# ceRNA Network of lncRNA/miRNA as Circulating Prognostic Biomarkers in Non-Hodgkin Lymphomas: Bioinformatic Analysis and Assessment of Their Prognostic Value in an NHL Cohort

**DOI:** 10.3390/ijms23010201

**Published:** 2021-12-24

**Authors:** Mara Fernandes, Herlander Marques, Ana Luísa Teixeira, Rui Medeiros

**Affiliations:** 1 Molecular Oncology and Viral Pathology Group, Research Center of IPO Porto (CI-IPOP)/RISE@CI-IPOP (Health Research Network), Portuguese Oncology Institute of Porto (IPO Porto)/Porto Comprehensive Cancer Center (Porto.CCC), 4200-072 Porto, Portugal; mara.aires.fernandes@ipoporto.min-saude.pt (M.F.); ana.luisa.teixeira@ipoporto.min-saude.pt (A.L.T.); 2 Research Department of the Portuguese League against Cancer Regional Nucleus of the North (LPCC-NRN), 4200-177 Porto, Portugal; 3 Faculty of Medicine, University of Porto (FMUP), 4200-319 Porto, Portugal; 4 Life and Health Sciences Research Institute (ICVS), School of Medicine, Campus de Gualtar, University of Minho, 4710-057 Braga, Portugal; herlander.marques@hb.min-saude.pt; 5 ICVS/3B’s—PT Government Associate Laboratory, 4805-017 Braga/Guimarães, Portugal; 6 Department of Oncology, Hospital de Braga, 4710-243 Braga, Portugal; 7 CINTESIS, Center for Health Technology and Services Research, Faculty of Medicine, University of Porto, 4200-450 Porto, Portugal; 8 ICBAS—Instituto de Ciências Biomédicas Abel Salazar, Universidade do Porto, 4050-513 Porto, Portugal; 9 Biomedical Research Center (CEBIMED), Faculty of Health Sciences of Fernando Pessoa University (UFP), 4249-004 Porto, Portugal

**Keywords:** non-hodgkin lymphoma, miRNA, lncRNA, ceRNA network, prognosis, biomarker

## Abstract

Research has been focusing on identifying novel biomarkers to better stratify non-Hodgkin lymphoma patients based on prognosis. Studies have demonstrated that lncRNAs act as miRNA sponges, creating ceRNA networks to regulate mRNA expression, and its deregulation is associated with lymphoma development. This study aimed to identify novel circulating prognostic biomarkers based on miRNA/lncRNA-associated ceRNA network for NHL. Herein, bioinformatic analysis was performed to construct ceRNA networks for hsa-miR-150-5p and hsa-miR335-5p. Then, the prognostic value of the miRNA–lncRNA pairs’ plasma levels was assessed in a cohort of 113 NHL patients. Bioinformatic analysis identified MALAT1 and NEAT1 as hsa-miR-150-5p and has-miR-335-5p sponges, respectively. Plasma hsa-miR-150-5p/MALAT1 and hsa-miR335-5p/NEAT1 levels were significantly associated with more aggressive and advanced disease. The overall survival and progression-free survival analysis indicated that hsa-miR-150-5p/MALAT1 and hsa-miR335-5p/NEAT1 pairs’ plasma levels were remarkably associated with NHL patients’ prognosis, being independent prognostic factors in a multivariate Cox analysis. Low levels of hsa-miR-150-5p and hsa-miR-335-5p combined with high levels of the respective lncRNA pair were associated with poor prognosis of NHL patients. Overall, the analysis of ceRNA network expression levels may be a useful prognostic biomarker for NHL patients and could identify patients who could benefit from more intensive treatments.

## 1. Introduction

Non-Hodgkin lymphomas (NHL) are a heterogenous class of lymphoproliferative malignancies, characterized by infiltration of lymphoid tissues [1]. The majority of NHL cases comprises the aggressive diffuse large B-cell lymphoma (DLBCL) and the indolent follicular lymphoma (FL), accounting for about 65% of all NHL cases [1]. Moreover, the latest GLOBOCAN data indicate that NHL represents the most common hematological malignancy worldwide, corresponding to approximately 3% of cancer diagnoses and cancer deaths [2]. The standard therapy regime for NHL treatment remains the anthracycline-containing chemotherapy (cyclophosphamide, doxorubicin, vincristine, prednisone—CHOP) combined with anti-CD20 agent, Rituximab (R-CHOP) [1]. However, despite the improvement in patients’ outcome after Rituximab introduction, approximately 20–50% of patients are refractory ab initio or ultimately relapse, with these patients presenting only a 20–40% 2-year overall survival rate [3,4,5]. In the past decade, the advance of gene-expression profiling and next-generation sequencing provided substantial insights about the etiology and the molecular background of the different entities comprising NHL, but the underlying molecular mechanisms still remain unclear. One of the main challenges in NHL management continues to be the ability to reliably stratify patients according to their risk of recurrence. Current clinical prognostic factors are associated with unpredictability in outcome within the individual risk groups, reflecting the biological heterogeneity of NHL, and emphasize the impending need of more precise and biologically based risk factors [6,7]. The main goal is to change from a clinical relapse to a molecular relapse evaluation in order to allow a more precise prognostic stratification, allowing early secondary therapeutic interventions.

Circulating non-coding RNAs, namely microRNAs (miRNAs) and long non-coding RNAs (lncRNAs), have emerged as a potential alternative to monitor the kinetics of the disease to be analyzed as “liquid biopsy” [8]. Since these molecules can be detected in a variety of biological fluids and due to their intrinsic stability, they represent a promising strategy as potential non-invasive diagnostic, prognostic, treatment response and disease monitoring biomarkers [9]. MiRNAs is the class of ncRNAs most studied over the years, especially due to their relevant biological function in gene regulation [10]. MiRNAs are characterized as small ncRNA with ~22 nucleotides of length, that function as gene regulators at the post-transcriptional level, through binding to the 3′ untranslated region (UTR) of a target mRNA, which results in their repression or degradation [11]. Dysregulated expression of miRNAs has been reported in lymphomas [12]. The expression levels of hsa-hsa-miR-27a, hsa-hsa-miR-142, hsa-miR-199b, hsa-miR-222, hsa-miR-302, hsa-miR-330, hsa-miR-425, and hsa-miR-519 seemed to be associated with OS of R-CHOP treated patients with DLBCL [13]. Recently, lncRNAs have emerged as a new level of gene expression regulation, playing an important role in lymphoma development [14]. LncRNAs are >200 nt long transcripts with no protein-coding capacity [15]. Moreover, lncRNAs regulate gene expression at multiple levels, by interacting not only with RNA but also with DNA and proteins [16]. Interestingly, Salmena et al. first proposed the hypothesis of “competing endogenous RNA (ceRNA) network”, where lncRNAs act as endogenous molecular sponges of miRNAs to regulate the expression of mRNAs [17]. Huang et al. reported that lncRNA LINC00857 regulates the hsa-miR-370-3p/CBX3 axis by competing for hsa-miR-370-3p binding, resulting in modulation of DLBCL cell proliferation and apoptosis [18]. Another recent study observed that lncRNA SNHG8 acts as a sponge to hsa-miR-335-5p, promoting proliferation while inhibiting apoptosis of DLBCL cells [19]. Even though most lncRNAs have not yet been characterized, the majority of them have shown great potential and clinical value [20,21].

It is clear that miRNAs and lncRNAs have a synergetic role in an intricated method to fine-tune gene expression; however additional studies are needed to further define the role of ncRNAs in clinical practice as potential new predictive and prognostic biomarkers. Hsa-miR-150-5p has been described as one of the players involved in B cell differentiation and as a potential non-invasive B cell lymphoma biomarker [22,23]. On the other hand, numerous studies showed that hsa-miR-335-5p is dysregulated in several cancers, such as breast cancer, lung cancer, colorectal cancer, and ovarian cancer [24,25,26,27]. Given the role of hsa-miR-335-5p in proliferation, apoptosis, migration, and invasion, this miRNA has attracted widespread attention as a potential prognosis biomarker. In fact, B cell CLL/lymphoma 2 like 2 (BCL-W or BCL2L2), which is an anti-apoptotic member of the Bcl-2 protein family, is identified as a possible target of hsa-miR-335-5p based on the results of bioinformatics analysis, indicating a potential role of this miRNA in NHL pathophysiology [28,29].

In the present study, we sought to analyze the potential of hsa-miR-150-5p and hsa-miR-335-5p as prognostic biomarkers, by studying their expression profile and their respective target lncRNAs in plasma samples of NHL patients.

## 2. Results

### 2.1. LncRNA-miRNA—mRNA Network Construction

**Figure 1:**
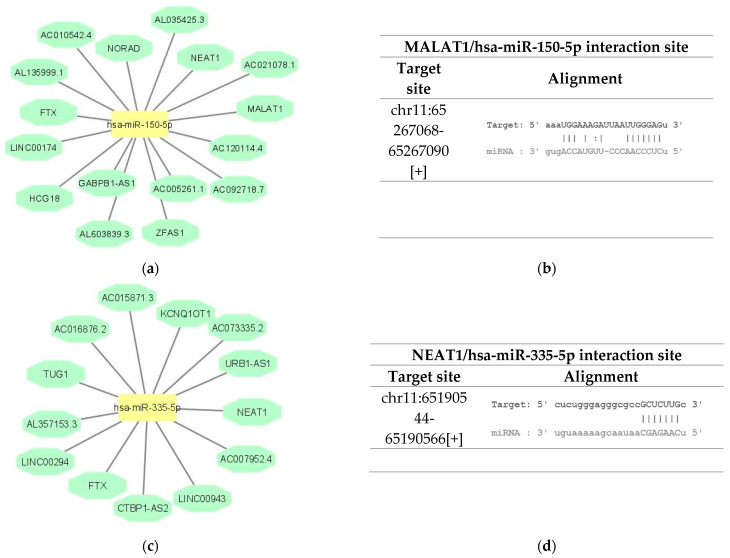
In silico analysis of the lncRNAs targeting hsa-miR-150-5p and hsa-miR-335-5p. (**a**) LncRNAs that target hsa-miR-150-5p according to StarBase database analysis; (**b**) details about the binding site of hsa-miR-150-5p on MALAT1, predicted by StarBase database; (**c**) lncRNAs that target hsa-miR-335-5p according to StarBase database analysis; (**d**) details about the binding site of hsa-miR-335-5p on NEAT1, predicted by StarBase database.

ceRNA is a well-known regulatory mechanism of lncRNAs. LncRNA sponges a variety of miRNAs to inhibit its expression and reduce the regulatory effect on the target mRNA [17]. The target recognition of hsa-miR-150-5p and hsa-miR-335-5p were analyzed via StarBase database and the miRNA–lncRNA pairs were identified. The analysis identified 17 lncRNAs targeting hsa-miR-150-5p, of which MALAT1 presented the higher CLIP Data evidence (Figure 1a,b). Concerning the analysis of the lncRNAs targeting hsa-miR-335-5p, 14 lncRNAs were identified, of which NEAT1 presented the higher CLIP Data evidence (Figure 1c,d). Therefore, both MALAT1 and NEAT1 were selected to be analyzed in plasma samples of NHL patients.

**Figure 2:**
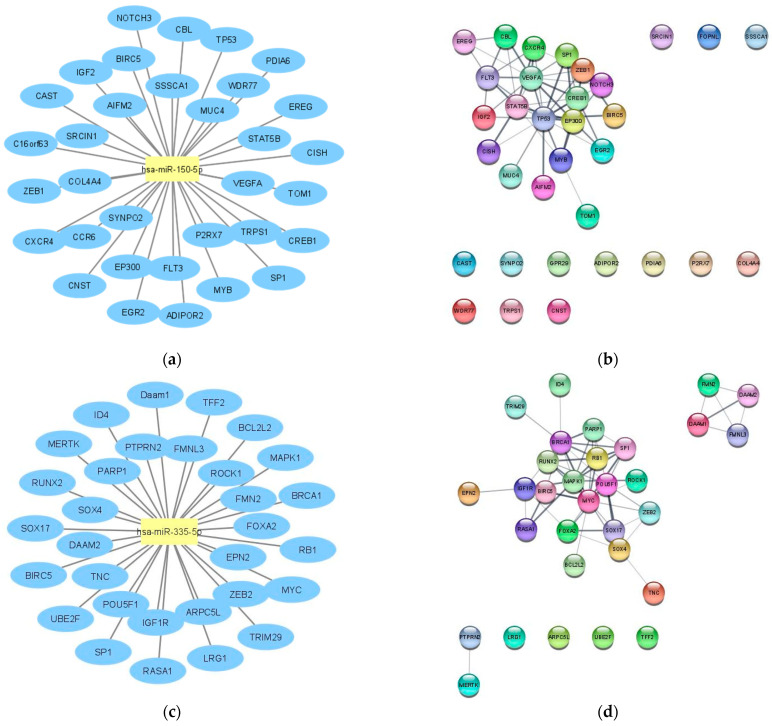
In silico analysis of the target mRNAs by hsa-miR-150-5p and hsa-miR-335-5p. (**a**) hsa-miR-150-5p target mRNAs according to miRTarBase database analysis; (**b**) target mRNAs of hsa-miR-150-5p organized by string interactions clusters; (**c**) hsa-miR-335-5p target mRNAs according to miRTarBase database analysis; (**d**) target mRNAs of hsa-miR-335-5p organized by string interactions clusters.

**Figure 3:**
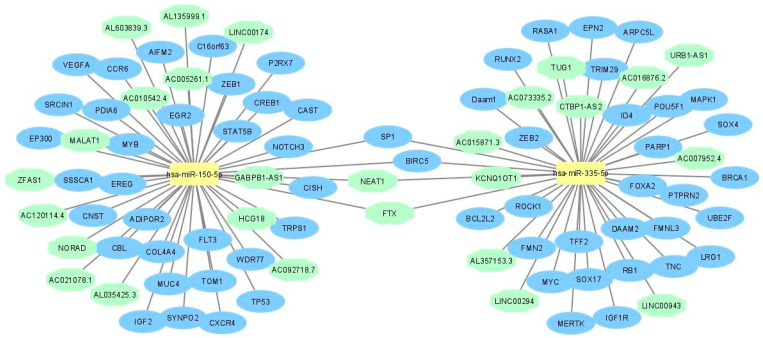
LncRNA–miRNA–mRNA networks related to hsa-miR-150-5p and hsa-miR-335-5p.

Then, miRTarBase database was employed to identify target mRNAs that are suppressed by hsa-miR-150-5p and hsa-miR-335-5p. According to miRTarBase analysis, the largest known online database of validated miRNA:mRNA interactions, we retrieved the list of mRNAs that were validated with strong evidence methods (Western blot, qRT-PCR or luciferase assay), which are listed in Figure 2a,c, in order to perform the functional annotation and enrichment analysis. To explore the biological impact, we analyzed the validated targets with the STRINGapp Protein Query from Cytoscape software. The identified targets were filtered into a protein–protein interaction (PPI) network with 33 nodes and 52 edges for hsa-miR-150-5p targets and 31 nodes and 62 edges for the hsa-miR-335-5p targets, presenting a significant enrichment (*p* = 4.17 × 10^−7^ and *p* = 1×10^−16^, respectively). We also applied a Markov clustering (MCL), which resulted in the clustering of the proteins according to their STRING interaction score (Figure 2b,d). Finally, we integrated the data from hsa-miR-150-5p and hsa-miR-335-5p identified targets and constructed the complete lncRNA–miRNA–mRNA network (Figure 3).

**Figure 4:**
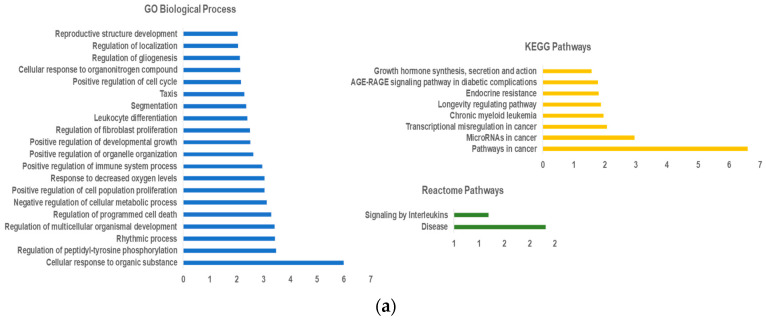

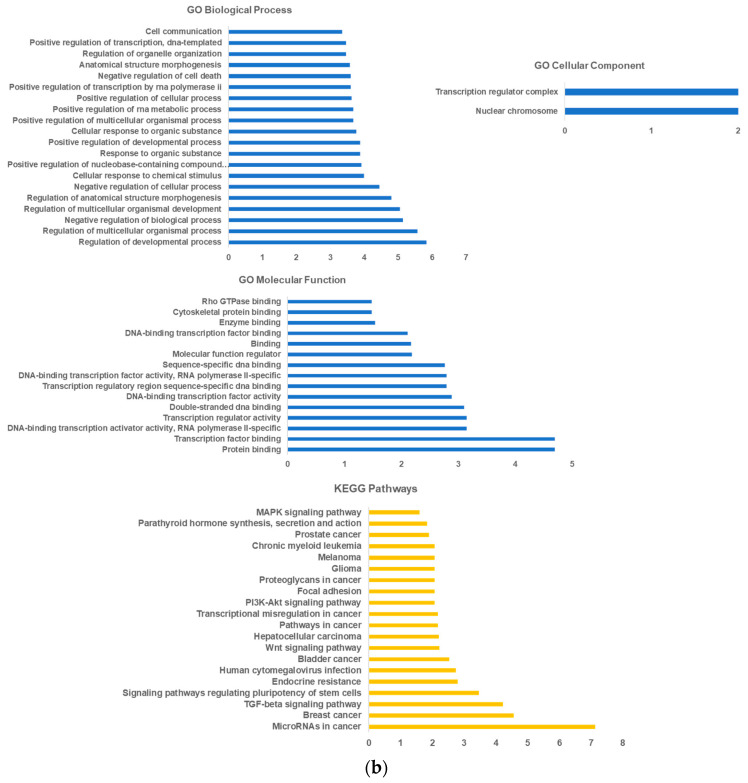
Enrichment analysis for hsa-miR-150-5p (**a**) and hsa-miR-335-5p targets (**b**).

For the functional enrichment analysis, we used an FDR threshold of *p* < 0.05, and the redundant terms were eliminated using a redundancy cutoff of 0.5, which resulted in a total of 54 enriched terms among the KEGG, Reactome and GO categories for hsa-miR-150-5p and 47 enriched terms for hsa-miR-335-5p. The enriched terms for each category are represented in Figure 4, representing only the top 20 enriched terms for GO Biological Process and KEGG pathways. Analyzing the functionally enriched terms for the hsa-miR-150-5p targets, we can observe that the majority of the targets are involved in major cancer pathways, including TP53, NOTCH3, SP1, STAT5B, EP300 and VEGFA, and they are associated with miRNA deregulation in cancer, all of which are well established involved factors in NHL development. In fact, concerning the GO molecular processes terms, we can observe that the enriched terms are associated with the regulation of cell proliferation and cell apoptosis, and the cellular response to organic substances. In the functional analysis of hsa-miR-335-5p targets through KEGG pathways, we could find PI3K/Akt and Wnt pathways, highly associated with miRNA deregulation, all central signaling pathways involved in the development and progression of NHL.

### 2.2. hsa-miR-150-5p, hsa-miR-335-5p, MALAT1 and NEAT1 Expression Levels in NHL Patients’ Plasma Samples

**Figure 5:**
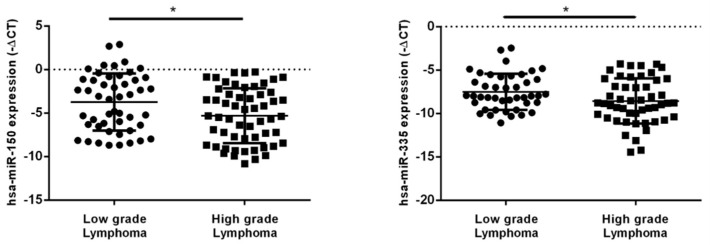
Expression levels of hsa-miR-150-5p and hsa-miR-335-5p in plasma samples of NHL patients’ groups according to lymphoma grade. Hsa-miR-150-5p and hsa-miR-335-5p presented lower plasma levels in patients with high grade lymphoma compared to patients with low grade lymphoma (*p* = 0.015 and *p* = 0.034, respectively). * *p* < 0.05.

We evaluated the plasma expression levels of hsa-miR-150-5p and hsa-miR-335-5p in 113 NHL patients by qRT–PCR. The expression levels of hsa-miR-150-5p and hsa-miR-335-5p in the plasma were significantly lower in patients with high grade lymphoma compared to low grade lymphoma (*p* = 0.015 and *p* = 0.034, respectively) (Figure 5).

**Figure 6:**
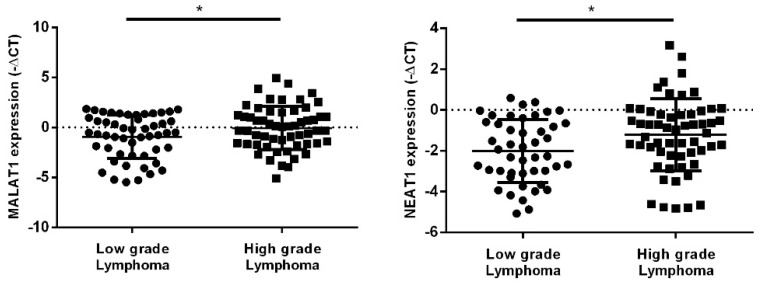
Expression levels of MALAT1 and NEAT1 in plasma samples of NHL patients’ groups according to lymphoma grade. MALAT1 and NEAT1 presented higher plasma levels in patients diagnosed with high grade lymphoma compared to low grade lymphoma (*p* = 0.035 and *p* = 0.018, respectively). * *p* < 0.05.

The plasma levels of MALAT1 and NEAT1 were higher in patients with high grade lymphoma compared to low grade lymphoma (*p* = 0.035 and *p* = 0.018, respectively) (Figure 6).

### 2.3. Association of miRNAs and lncRNAs Plasmatic Levels and the Clinicopathological Characteristics of NHL Patients

In this study, the mean of each transcript expression levels of the 113 patients were used as the threshold, and patients were divided into a high-level expression group (with an expression level above the mean expression level) and a low-level expression group (with an expression level below the mean). The association between the plasma expression levels and the clinicopathological features of the patients is summarized in Supplementary Table S1. The analysis revealed that low levels of miRNA-150 are associated with III/IV stages (*p* = 0.015), high levels of LDH (*p* = 0.020) and higher IPI and FLIPI scores (*p* = 0.043 and *p* = 0.034, respectively). Lower expression levels of hsa-miR-335-5p were associated with III/IV stages (*p* = 0.010), higher ECOC (*p* = 0.037) and higher IPI score (*p* = 0.012). Moreover, no correlation between age and gender and the expression levels of both miRNAs was found. Concerning the expression levels of MALAT1, high levels of the transcript are associated with advanced stages (*p* = 0.018) and higher IPI and FLIPI scores (*p* = 0.034 and *p* = 0.025, respectively). Similarly, high levels of NEAT1 are also associated with III/IV stages (*p* = 0.018), presence of B symptoms (*p* = 0.038) and higher IPI score (*p* = 0.032).

### 2.4. miRNAs and lncRNAs Impact on Overall Survival and Progression-Free Survival of NHL Patients

**Figure 7:**
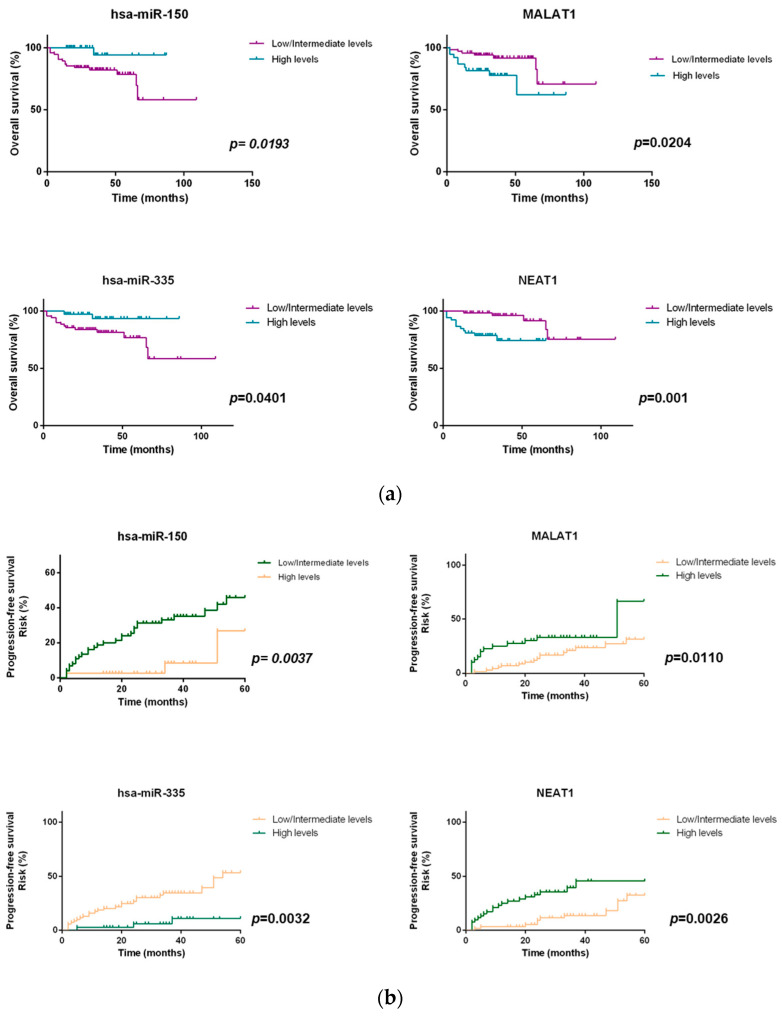
OS (**a**) and PFS (**b**) of NHL patients according to plasma levels of hsa-miR-150-5p, hsa-miR-335-5p, MALAT1 and NEAT1.

Patients were divided in terciles according to the transcript levels using the −ΔCt values of each miRNA and lncRNA (high, intermediate, and low levels), in order to analyze the association between OS and PFS of NHL patients and the expression levels of the transcripts. The Kaplan–Meier estimate and log-rank tests revealed that NHL patients with lower levels of hsa-miR-150-5p and hsa-miR-335-5p, and higher levels of MALAT1 and NEAT1 present worse OS (hsa-miR-150-5p: *p* = 0.019; hsas-miR-335-5p: *p* = 0.040; MALAT1: *p* = 0.020; NEAT1: *p* = 0.001) and worse PFS (hsa-miR-150-5p: *p* = 0.004; hsa-miR-335-5p: *p* = 0.003; MALAT1: *p* = 0.011; NEAT1: *p* = 0.003) than those with higher levels of hsa-miR-150-5p and hsa-miR-335-5p and lower levels of MALAT1 and NEAT1 (Figure 7).

**Figure 8:**
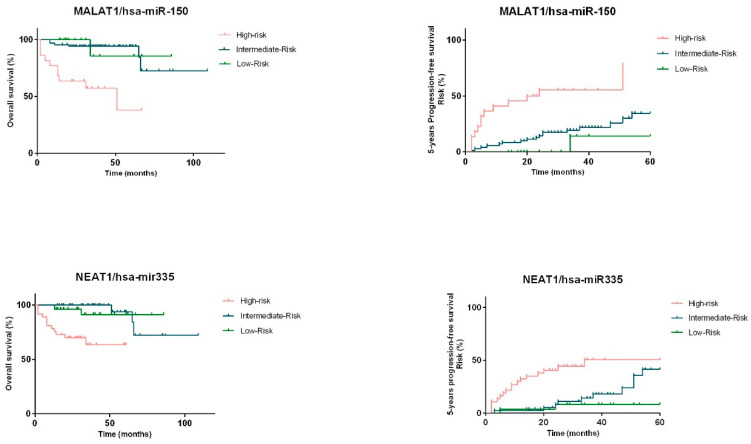
OS and PFS of NHL patients according to plasma levels of miRNA/lncRNA pairs (*p* < 0.001).

Then, three groups were established considering the combination of the plasma levels of each ceRNA pair, hsa-miR-150-5p/MALAT1 plasma levels and hsa-miR-335-5p/NEAT1 plasma levels which allowed the definition of high-, intermediate- and low-risk groups (Table 1). For the analysis of the hsa-miR-150-5p-MALAT1 pair, the low-risk group was constituted of patients combining high expression of hsa-miR-150-5p and low expression of MALAT1. The intermediate-risk group combined both patients with high hsa-miR-150-5p and high MALAT1 expression and patients with low hsa-miR-150-5p and low MALAT1 expression. The high-risk group combined patients with a low expression of hsa-miR-150-5p and high expression of MALAT1. Concerning ceRNA pair hsa-miR-335-5p-NEAT1, the low-risk group was constituted of patients combining high expression of hsa-miR-335-5p and low expression of NEAT1. The intermediate-risk group combined both patients with high hsa-miR-335-5p and high NEAT1 expression and patients with low hsa-miR-335-5p and low NEAT1 expression. The high-risk group combined patients with a low expression of hsa-miR-335-5p and high expression of NEAT1. Given the results, we observed that the significance of the prognostic value is improved when expression levels of the miRNAs were combined with the respective lncRNA pair (*p* < 0.001) (Figure 8).

Multivariate analysis was conducted with significant clinical parameters associated with patients’ prognosis and revealed that low expression of hsa-miR-150-5p and high expression of MALAT1 were independent poor prognostic factors of OS and PFS (OS: *p* = 0.020 and *p* = 0.026; PFS: *p* = 0.027 and *p* = 0.021, respectively) (Supplementary Table S2). Similarly, regarding the hsa-miR-335-5p-NEAT1 ceRNA pair, low levels of hsa-miR-335-5p and high levels of MALAT1 were observed to be also independent poor prognostic factors of OS and PFS (OS: *p* = 0.037 and *p* = 0.002; PFS: *p* = 0.009 and *p* = 0.040, respectively) (Supplementary Table S3). Patients with low hsa-miR-150-5p/high MALAT1 and low hsa-miR-335-5p levels/high NEAT1 levels present a higher risk of death and disease progression compared to patients with high levels of hsa-miR-150-5p/low levels MALAT1 and high levels of hsa-miR-335-5p/low levels NEAT1.

## 3. Discussion

Despite the improvements in NHL patients’ overall survival, there is still an unpredictability factor in the outcome within individual risk groups, with a considerable percentage of patients being refractory ab initio or ultimately relapse, with poor 2-year overall survival rates [3,4,5]. This unpredictability in patients’ outcome can be the reflection of the pathological and molecular heterogeneity of NHL. The IPI systems are the current methodology used to infer risk stratification and predict the NHL patients’ outcome. However, the IPI systems do not consider factors inherent to the molecular heterogeneity of patients, leading to the critical need of identifying additional molecular prognostic biomarkers. In the last decade, several studies have demonstrated the contribution of miRNAs to lymphoma development, implying their potential as novel biomarkers in cancer diagnosis and prognosis [14]. More recently, a new class of ncRNA, the lncRNAs, has emerged also as important regulators of gene expression, and their deregulation has been implicated in the initiation and progression of cancer, showing great potential as predictive biomarkers. However, the majority of studies focused on solid tumors [30,31,32].

Given the ability of lncRNA to sponge miRNAs and consequently inhibiting their inhibitory function over target mRNAs, in this study, we investigated the prognostic value of lncRNAs and miRNAs by analyzing lncRNAs–miRNAs pairs’ expression in plasma samples of NHL lymphomas. According to our results, hsa-miR-150-5p, hsa-miR-335-5p, MALAT1 and NEAT1 were differentially expressed in patients with high grade lymphomas when compared to low grade lymphoma patients, indicating their involvement in the pathogenesis and prognosis of NHL. Specifically, lower levels of hsa-miR-150-5p and hsa-miR-335-5p and higher levels of MALAT1 and NEAT1 were associated with worse clinicopathologic characteristics, such as higher clinical stage and higher IPI/FLIPI scores and predicted poor prognosis and poor survival rates in NHL patients.

According to our bioinformatics analysis using StarBase database, the lncRNA MALAT1 was identified as a direct target of hsa-miR-150-5p. Interestingly, MALAT1 was shown to be involved in the maintenance of early-stage hematopoietic cell proliferation potential [33]. During B cell activation, MALTA1 is differentially expressed and was identified as a AID target, which is involved in both somatic hypermutation and class-switch recombination in activated B cells [34]. MALAT1 can regulate gene expression transcriptionally, by acting as a scaffold on interchromatin clusters, for example by interacting with EZH2 and SUZ12, two components of the PRC2 complex. In mantle cell lymphoma, MALAT1 was found upregulated and associated with advanced disease stage and lower overall survival, similarly to our obtained results. Wang et al. reported that overexpressed MALAT1 interacts with EZH2, highly expressed in B-cell lymphomas, facilitating the complex binding to gene promoters which results in suppression of p21 and p27 expression and increase c-Myc expression [35,36]. Furthermore, it was shown that MYC binds to the upstream region of has-miR-150 leading to its expression repression [37]. Intriguingly, MYC and EZH2 create a positive loop that constantly leads to MYC and EZH2 overexpression, and both act together to silence tumor suppressor miRNAs in aggressive lymphoma cells, such as hsa-miR-150 [37]. On the other hand, MALAT1 can also regulate gene expression post-transcriptionally by acting as a ceRNA sequestering miRNAs. For example, in acute myeloid leukemia, MALAT1 regulates cells migration, proliferation and apoptosis, by releasing CXCR4 from the regulatory inhibition of hsa-miR-146 [38]. In multiple myeloma, MALAT1 regulates proliferation and adhesion of multiple myeloma cells via hsa-miR-181-5p/Hippo-YAP axis. Moreover, a study by Gu et al. reported that MALAT1 is overexpressed in multiple myeloma, and regulates cell proliferation, apoptosis, and cell-cycle through the ceRNA network involving the hsa-miR-509-5p/Foxp1 pathway [39]. Very recently, MALAT1 was reported to directly bind to hsa-miR-150, releasing their suppressive effect on the target mRNAs, promoting cell proliferation and inhibiting apoptosis [40,41]. Therefore, we propose that MALAT1 ensures downregulation of hsa-miR-150, required for development and progression of NHL cells by two mechanisms. Firstly, MALAT1 induces transcriptional inhibition of hsa-miR-150 by EZH2/MYC axis. Secondly, MALAT1 ensures the maintenance of low hsa-miR-150 levels by directly sponging it, inhibiting its repressive function on target mRNAs.

We also constructed hsa-miR-150-5p network to explore their role in NHL prognosis. Using a bioinformatics approach, we identified a central hsa-miR-150-related PPI network linked to MYB, TP53, SP1, STAT5B, NOTCH3, EP300 and CREB1. This PPI network was enriched in cell proliferation and cell cycle regulation, regulation of apoptosis, regulation of immune system pathways, and act as central players in cancer pathogenesis as demonstrated in the KEGG pathway analysis. In fact, the identified members of the hsa-miR-150-related PPI network have been associated with NHL development and progression. A top predicted target of hsa-miR-150-5p was MYB, a transcription factor and well-established regulator of the hematopoietic cell development and proliferation, with key target genes involved in cell cycle, cell proliferation and growth, differentiation, and survival, such as MYC, CCNB1, BCL2, BIRC5, and CDK1 [42,43,44,45,46,47]. In summary, the overall results show an intricate network involving MALAT1-EZH2-MYC-miR-150-MYB. Specifically, we hypothesize that upregulated MALAT1 concomitantly with EZH2 induces MYC expression, which binds to the hsa-miR-150 promoter region inhibiting its expression. Moreover, MALAT1 further ensures inhibition of hsa-miR-150 function post-transcriptionally by sponging hsa-miR-150. Thus, by downregulating hsa-miR-150 both transcriptionally and post-transcriptionally, MALAT1 unleashes MYB from hsa-miR-150-mediated repression resulting in high cell proliferation.

On the other hand, NEAT1 was identified as one of the lncRNA targets of hsa-miR-335-5p. NEAT1 has been identified as a cancer driver, playing a role in tumor initiation and progression, and its expression was found deregulated in several cancers [48,49,50,51,52,53,54]. Given the role of NEAT1 in cancer, several studies have been trying to unravel the molecular mechanisms regulating its expression and its targets. Recently, a study by Liu et al. reported that NEAT1 is transcriptionally regulated by MYC, inducing its transcription [55]. Interestingly, a study by Zhu et al. demonstrated that NEAT1 regulates chromatin remodeling through increasing acetylation levels at MYC promoter, inducing its expression, in colorectal cancer [56]. Growing evidence has demonstrated that NEAT1 can function as a ceRNA by sponging several miRNAs. In nasopharyngeal carcinoma NEAT1 targets hsa-miR-34a-5p which in turn activates Wnt/β-catenin signaling promoting tumor cell proliferation, migration, invasion, and EMT [57]. Ding et al. reported that by negatively regulating hsa-miR-34a-5p expression, NEAT1 upregulates BCL-2 expression resulting in promotion of cell proliferation and inhibition of apoptosis [58]. In breast cancer, NEAT1 serves as a ceRNA to modulate ZEB1 function by sponging hsa-miR-448, promoting cancer progression [59]. Concerning cervical cancer, hsa-miR-133a appears downregulated by NEAT1, which in turn results in upregulation of SOX4 and cervical cancer progression [60]. In multiple myeloma, NEAT1 forms a positive feedback loop with SP1, in which NEAT1 induces SP1 expression by sequestering hsa-miR-29b-3p, and SP1 targets NEAT1 promoter region inducing NEAT1 transcription, and collectively promoting tumor cell survival [61]. NEAT1 was found increased in T-cell acute lymphoblastic leukemia, in which it upregulates NOTCH1 expression via sponging hsa-miR-146b-5p, and in multiple myeloma in which via inhibition of hsa-miR-214 it promotes M2 macrophage polarization by inducing the expression and release of B7-H3, resulting in the activation of JAK2/STAT3 signaling [62,63].

Several studies have been unraveling the role of hsa-miR-335-5p as a tumor suppressor in several cancers, such as inhibiting cell invasion and metastasis in thyroid cancer, epithelial–mesenchymal transition in NSCLC, cell proliferation and metastasis in osteosarcoma, and cancer cell growth in colorectal cancer [64,65,66,67]. Moreover, hsa-miR-335-5p interplay with lncRNAs has also been described to play a regulatory role in the development and progression of osteosarcoma, cervical cancer, and bladder cancer [68,69,70]. In fact, NEAT1 was shown to sponge hsa-miR-335-5p, releasing ROCK1 from its suppression, which promotes cell proliferation, migration, and invasion in gastric cancer [71]. Moreover, by sequestering hsa-miR-335-5p, NEAT1 induces AKT phosphorylation and c-MET expression in hepatocellular carcinoma cells [72]. However, whether hsa-miR-335-5p regulates NHL remains to be further investigated. A recent study indicates a possible role of hsa-miR-335-5p in DLBCL cells via SNHG8 sponging hsa-miR-335-5p, promoting proliferation and inhibiting apoptosis [19].

Using a bioinformatics analysis, we identified an hsa-miR-335-related PPI network linked to proteins such as MYC, ZEB2, SP1, SOX4, and BCL2L2. This PPI network was enriched in negative regulation of cell death, positive regulation of cell proliferation and deregulation of miRNAs in cancer pathogenesis as demonstrated in the KEGG pathway analysis. Interestingly, not only NEAT1 and hsa-miR-335-5p were shown to directly interact with each, in which NEAT1 functions as hsa-miR-335-5p sponge, but also there is an overlap of downstream targets, such as MYC, ZEB2, SP1, SOX4, and BCL2L2. Therefore, we hypothesize a potential involvement of NEAT1-mediated suppression of hsa-miR-335-5p leading to promotion of proliferation signaling networks and inhibition of apoptosis with potential involvement in NHL progression.

Only very recently, the interplay between lncRNAs and miRNAs is being unraveled, and few studies have reported the deregulation of lncRNA–miRNA in NHL tissue samples and cell lines, and their abnormal expression levels were associated with poor prognosis. Our study introduces circulating miRNAs and their associated lncRNAs as novel complementary biomarkers in NHL prognosis. Despite the novelty of our study by introducing the analysis of ceRNA network components as useful circulating biomarkers for NHL prognosis, our study presents a few limitations. It would be interesting to also evaluate the expression of mRNAs related to the identified ceRNA networks and analyze their clinical value. Moreover, future studies should focus on validating and clarifying the biological function of the identified circulating transcripts, by, for example, performing in vitro studies to modulate the expression of the transcripts and investigate their influence on tumor cell properties (e.g., cell proliferation and cell apoptosis).

## 4. Materials and Methods

### 4.1. Construction of CeRNA Regulatory Network and Functional Analysis

First, miRNA–lncRNA interactions were evaluated using the StarBase database (https://starbase.sysu.edu.cn/ (accessed on 13 January 2020)) with the default parameters (clade: mammal, genome: human, assembly: hg19, number of supporting experiments: ≥3, pan-cancer ≥ 2) [73].

Next, miRTarBase (https://bio.tools/mirtarbase (accessed on 20 June 2021)) (only validated by strong evidence methods) was used to retrieve miRNA-targeted mRNAs [74]. To analyze the protein–protein interaction (PPI) networks the Search Tool for the Retrieval of Interacting Genes (STRING) database was used. The STRINGapp of the Cytoscape software (v3.8.2, Cytoscape Consortium, San Diego, CA, USA) was used to construct and visualize the protein interaction network of the selected target genes. The functional enrichment analysis of Gene Ontology (GO), Kyoto Encyclopedia of Genes and Genomes (KEGG) and Reactome pathways was performed using the STRING enrichment analysis tool. According to the Jaccard index, the enrichment results were filtered, and redundant terms were removed. The interaction networks were constructed using Cytoscape visualization software (https://cytoscape.org/ (accessed on 22 September 2021)) [75].

### 4.2. Study Population

The study included 113 patients diagnosed with B-cell NHL (high grade lymphomas versus low grade lymphomas), from Caucasian ethnicity, older than 18 years and without known familiar cancer history. Patients were admitted and treated at a Portuguese Hospital between January 2016 and June 2020. Clinical, laboratory and pathology data were collected by reviewing the medical records of each patient. Patient’s clinical information included gender, age, international prognostic index (IPI) score, B symptoms, serum LDH levels, Ann Arbor stage, Eastern Cooperative Oncology Group (ECOG) performance status and treatment regime and treatment response and are summarized in Table 2. Patients who had central nervous system involvement, previous immunosuppressive treatments, or who were associated with HIV or HBV infections were excluded. This study was conducted according to the principles of the Helsinki declaration and ethics committee of the hospital. All individuals signed written informed consent in order to participate in the study.

### 4.3. RNA Extractions and qPCR

Blood samples were taken at baseline before starting therapy. Peripheral blood samples were centrifuged for 5 min at 3000 rpm at room temperature to separate the plasma fraction, followed by preparation of platelet-free plasma (PFP) by centrifugating at 2500× *g* for 15 min at 16 °C. Supernatant was then transferred into a new centrifuge tube and centrifuged again at 16 °C 2500× *g* for 15 min [34]. Supernatant was aliquoted and stored at −80 °C until use. MiRNA isolation from PFP samples were performed using the GRS microRNA kit (Grisp^®^) according to laboratory procedures, and total RNA isolation was performed using Plasma/Serum RNA Purification Kit (Norgen^®^ Biotek Corp. Thorold, ON, Canada). MiRNA and RNA concentration and purity were measured using the NanoDrop Lite spectrophotometer (Thermo Scientific^®^, Waltham, MA, USA). Synthesis of cDNA was carried out using TaqMan^®^ MicroRNA Reverse Transcription kit and TaqMan^®^ MicroRNA assay (Applied Biosystems^®^, Waltham, MA, USA). cDNA was synthesized from lncRNA using the High-Capacity cDNA Reverse Transcription Kit (Applied Biosystems^®^, Waltham, MA, USA) in accordance with manufacturer’s instructions. Quantification of the miRNA and lncRNA expression levels were performed by qPCR using a StepOneTM qPCR Real-Time PCR machine, using 1× TaqMan^®^ Gene Expression Master mix (Thermo Fisher Scientific^®^) and 1× probes TaqMan^®^MicroRNA Assays (hsa-miR-150-5p: 000473 and hsa-miR-335-5p: 002185) and using TaqMan^®^ Noncoding RNA assays (MALAT1: Hs00273907 and NEAT1: Hs01008264_s1) (Thermo Fisher Scientific^®^). Expression levels of miRNAs were normalized to hsa-miR-16 (hsa-miR-16: 000391) and expression levels of lncRNAs were normalized to GAPDH (GAPDH: Hs99999905_m1) endogenous control. Two technical replicates were made for each sample. The amplification conditions were a holding stage 95 °C for 20 s, followed by 45 cycles of 95 °C for 1 s, and 60 °C for 20 s. Data analysis was made using StepOneTM Sofware v2.2 (Applied Biosystems^®^, Foster City, CA, USA) with the same baseline and threshold set for each plate.

### 4.4. Statistical Analysis

Statistical analysis was performed using SPSS (version 26.0; IBM Company, Chicago, IL, USA) and GraphPad Prism (version 7.0; GraphPad Software, Inc. San Diego, CA, USA) software. The 2-ΔΔCt method (Livak method) and the Student’s t-test or Mann–Whitney U test were used to evaluate statistical differences in the normalized expression of the miRNAs and lncRNAs. For association analyses between transcripts’ expression levels and clinicopathological features was used Chi-square test. Overall survival (OS) and progression-free survival (PFS) curves were generated using Kaplan–Meier method and compared using the log-rank test. OS time was determined from the date of diagnosis to the date of mortality or the last follow-up. PFS time was determined from the date of diagnosis to the date of disease progression, recurrence, mortality or last follow-up. Cox regression was used to analyze the prognostic value of the miRNAs/lncRNAs expression levels on the progression-free and overall survival. *p* < 0.05 was considered statistically significant.

## 5. Conclusions

Analysis of plasma levels of hsa-miR-150-5p/MALAT1 and hsa-miR-335-5p/NEAT1 pairs could be useful novel non-invasive prognostic biomarkers for NHL. Plasma hsa-miR-150-5p/MALAT1 and hsa-miR-335-5p/NEAT1 levels were associated with patient clinical outcome, where patients with low hsa-miR-150-5p/high MALAT1 and low hsa-miR-335-5p/high NEAT1 present worse overall survival and progression-free survival. Therefore, our results could shed light on the development of precision medicine with the identification of miRNA–lncRNA profiles that permits the identification of risk patients, improving the management of NHL patients.

## Figures and Tables

**Table 1:** Definition of high-, intermediate- and low-risk groups considering the combination of the plasma levels of each ceRNA pair, hsa-miR-150hsa-miR-150-5p/MALAT1 plasma levels and hsa-miR-335hsa-miR-335-5p/NEAT1 plasma levels.

Groups	hsa-miR-150-5p-MALAT1	hsa-miR-335-5p-NEAT1
Low-risk	↑hsa-miR-150 +↓MALAT1	↑hsa-miR-335 +↓NEAT1
Intermediate-risk	↑hsa-miR-150 +↑MALAT1 ↓hsa-miR-150 +↓MALAT1	↑hsa-miR-335 +↑NEAT1 ↓hsa-miR-335 +↓NEAT1
High-risk	↓hsa-miR-150 +↑MALAT1	↓hsa-miR-335 +↑NEAT1

**Table 2:** Patients’ clinicopathologic characteristics.

Clinical-Pathological Characteristics	N (%) N = 113
Age	
≤60 years	53 (46.9%)
>60 years	60 (53.1%)
Gender	
Female	57 (50.4%)
Male	56 (49.6%)
Grade	
Low (indolent)	55 (48.7%)
High (aggressive)	58 (51.3%)
Subtype of NHL	
Follicular	40 (35.4%)
Diffuse large B-cell	58 (51.3%)
Marginal Zone	15 (13.3%)
Stage	
I/II	43 (38.1%)
III/IV	70 (61.9%)
LDH serum levels	
Normal	67 (59.3%)
High	45 (39.8%)
Unknown	1 (0.9%)
ECOG	
0–1	97 (85.8%)
≥2	14 (12.4%)
Unknown	2 (1.8%)
B symptoms	
Absent	80 (70.8%)
Present	33 (29.2%)
IPI Score (high grade tumors)	
Low-risk (0–1)	17 (29.3%)
Intermediate-risk (2–3)	25 (43.1%)
High-risk (4–5)	14 (24.1%)
Unknown	2 (3.4%)
FLIPI score (low grade tumors)	
Low-risk (0–1)	18 (32.7%)
Intermediate-risk (2)	18 (32.7%)
High-risk (3, 4, 5)	19 (34.5%)
BM involvement	
Negative	81 (71.7%)
Positive	32 (28.3%)

## Supplementary Materials

The following supporting information can be downloaded at: www.mdpi.com/article/10.3390/ijms23010197/s1
