# Supplementary material for: ceRNA Network of lncRNA/miRNA as Circulating Prognostic Biomarkers in Non-Hodgkin Lymphomas: Bioinformatic Analysis and Assessment of Their Prognostic Value in an NHL Cohort
Source: Int J Mol Sci. 2021 Dec 24;23(1):201. doi: 10.3390/ijms23010201 (PMC8745130; doi:10.3390/ijms23010201)
Supplement: Supplementary file 1 [file ijms-23-00201-s001.zip › ijms-1519925-supplementary.pdf]

Supplementary Materials

Table S1. The correlation between the plasma levels of hsa-miR-150-5p, hsa-miR-335-5p, MALAT1 and NEAT1 and the clinicopathological characteristics of NHL patients.

**Characteristics**	**hsa-miR-150-5p**				**hsa-miR-335-5p**			
	**Evaluable (n)**	**Low levels (n)**	**High levels (n)**	**P**	**Evaluable (n)**	**Low levels (n)**	**High levels (n)**	**P**
**Gender**				0.708				0.423
Female	57	27	30		52	25	27	
Male	55	28	27		50	28	22	
**Age, years**				0.556				
≤60	53	24	29		46	21	25	0.248
>60	59	30	29		56	32	24	
**Stage**				**0.015**				**0.010**
I/ II	42	14	28		38	13	25	
III / IV	70	40	30		64	39	25	
**LDH serum levels**				**0.020**				0.469
Normal	67	26	41		59	28	31	
High	44	27	17		42	23	19	
**ECOG**				0.223				**0.037**
0-1	96	45	51		87	40	47	
≥ 2	14	9	5		13	10	3	
**B symptoms**				0.247				0.344
No	79	36	43		71	34	37	
Yes	33	19	14		31	18	13	
**IPI Score**				**0.043**				**0.012**
0-2	28	9	19		26	9	17	
3-5	27	16	11		26	18	8	
**FLIPI score**				**0.034**				0.059
0-2	36	12	24		31	12	19	
3-5	19	12	7		18	12	6	
**BM involvement**				0.905				0.458
Negative	80	39	41		72	35	37	
Positive	32	16	16		30	17	13	

	**MALAT1**				**NEAT1**			
	**Evaluable (n)**	**Low levels (n)**	**High levels (n)**	**P**	**Evaluable (n)**	**Low levels (n)**	**High levels (n)**	**P**
**Gender**				0.332				0.386
Female	53	23	30		54	30	24	
Male	55	29	26		53	25	28	
**Age, years**				0.586				0.908
≤60	49	25	24		50	26	24	
>60	59	27	32		57	29	28	
**Stage**				**0.018**				**0.018**
I/ II	42	22	17		41	14	25	
III / IV	66	24	42		66	28	38	
**LDH serum levels**				0.102				0.250
Normal	63	33	30		61	27	34	
High	44	16	28		45	20	25	
**ECOG**				0.054				0.055
0-1	93	48	45		91	51	40	

≥ 2	13	3	10		14	4	10	
**B symptoms**				0.063				**0.038**
No	76	41	76		74	43	31	
Yes	32	11	21		33	12	21	
**IPI Score**				**0.034**				**0.032**
0-2	29	16	13		29	18	11	
3-5	26	7	19		27	9	18	
**FLIPI score**				**0.025**				0.539
0-2	33	20	13		32	18	14	
3-5	18	5	13		17	8	9	
**BM involvement**				0.347				0.206
Negative	79	38	41		78	43	35	
Positive	29	11	18		29	12	17	

Table S2. The results of the multivariate analysis for overall survival and 5-years progression free survival regarding the has-miR-150-5p/MALAT1 pair.

**Characteristic**	**OS**		**5-year PFS**	
	**HR (95% CI)**	**P**	**HR (95% CI)**	**P**
Age (≤60y vs >60y)	2.262 (0.703-7.281)	0.171	1.021 (0.396-2.635)	0.066
Lymphoma grade (Low vs High)	4.396 (1.240-8,581)	0.022	2.743 (1.081- 6.963)	0.034
B symptoms (Absent vs Present	2.941 (1.089-7.943)	0.033	1.803 (0.523-2.993)	0.136
Stage (I/II vs III/IV)	3.654 (1.276-7.020)	0.028	3.822 (1.280-5.413)	0.016
ECOG (0-1 vs ≥2)	2.827 (0.904-5.838)	0.074	1,958 (1.240-7.284)	0.055
LDH levels (normal vs high)	3.057(1.030-9.070)	0.044	1.760 (0.814-3.805)	0.151
hsa-miR-150-5p levels (Low/Inter vs. High)	0.170 (0.038-0.761)	0.020	0.239 (0.067-0.850)	0.027
MALAT1 levels (Low/Inter vs. High)	3.213 (1.153-8.955)	0.026	2.343 (1.137-4.828)	0.021

Table S3. The results of the multivariate analysis for overall survival and 5-years progression free survival regarding the has-miR-335-5p/NEAT1 pair.

**Characteristic**	**OS**		**5-year PFS**	
	**HR (95% CI)**	**P**	**HR (95% CI)**	**P**
Age (≤60y vs >60y)	3.288 (0.738-14.651)	0.118	1.556 (0.593-4.081)	0.369
Lymphoma grade (Low vs High)	3.735 (1.101-8.672)	0.035	3.929 (1.298-7.896)	0.015
B symptoms (Absent vs Present)	1.212 (0.332-4.433)	0.070	1.649 (0.772-3.522)	0.196
Stage (I/II vs III/IV)	3.560 (0.686-8.487)	0.031	3.864 (1.212-6.322)	0.022
ECOG (0-1 vs ≥2)	3.247 (0.9908-11.685)	0.070	2.902 (0.942-8.942)	0.064
LDH levels (normal vs high)	1.483 (0.444-4.958)	0.522	1.962 (0.813-4.739)	0.135
hsa-miR-335-5p levels (Low/Inter vs. High)	0.197 (0.043-0.909)	0.037	0.188 (0.53-0.663)	0.009
NEAT1 levels (Low/Inter vs. High)	8.694 (2.220-14.046)	0.002	2.543 (1.043-6.201)	0.040
